# Intranasal vaccines adjuvanted with Nexavant demonstrate robust protective efficacy by inducing both mucosal and systemic immunity in a murine model

**DOI:** 10.3389/fimmu.2025.1745319

**Published:** 2026-01-16

**Authors:** Kwang Hyun Ko, Hyun Shik Bae, So Min Lee, Somin Park, Seung Hyun Han, Jun Heo, Jinil Kim, Yerim Cho, Dong-Ho Kim, Seung Bin Cha

**Affiliations:** 1 Research and Development (R&D) Center, NA Vaccine Institute, Seoul, Republic of Korea; 2 Department of Oral Microbiology and Immunology, and Dental Research Institute, School of Dentistry, Seoul National University, Seoul, Republic of Korea; 3 Vaccine Research and Development (R&D) Team, Central Institute, Il Yang Pharmaceutical, Yongin, Republic of Korea; 4 Vaccine Research and Development (R&D) Center, Eubiologics Co., Ltd, Chuncheon, Republic of Korea

**Keywords:** intranasal vaccine, mucosal immunity, systemic immunity, TLR3 agonist, type I interferon, vaccine adjuvant

## Abstract

Mucosal vaccines offer the advantage of inducing immunity at pathogen entry sites; however, concerns about safety and limited efficacy have hindered the widespread use of viral-vectored intranasal vaccines. Nexavant (NVT), a well-defined TLR3 agonist, was investigated as a non-viral mucosal adjuvant. NVT was formulated with commercial subunit and polysaccharide–protein conjugate antigens and administered intranasally to mice. NVT-adjuvanted vaccines elicited robust mucosal IgA and systemic IgG responses, enhanced antigen-specific CD4^+^ T cell activation, and conferred strong protection against high-dose influenza virus challenge. Antigen-specific mucosal IgA was detected not only in nasal washes but also in distal mucosal sites such as saliva, vaginal washes, and feces, indicating broad mucosal immune crosstalk. These immune responses were abolished in IFNAR1^-^/^-^ mice, demonstrating a critical role for type I interferon signaling in NVT’s mechanism of action. The adjuvant was effective across diverse antigen types and demonstrated a favorable safety profile. These findings support NVT as a promising mucosal adjuvant platform for next-generation intranasal vaccines.

## 1 Introduction

The mucous membrane is a tissue that lines the inner surfaces of various cavities in the body. Since the respiratory, digestive, and urinary organs are in direct contact with the outside environment, the mucous membrane serves as the first line of defense against invading pathogens. Most pathogens penetrate through the mucosa, so developing immunity in this area is crucial for effectively combating these invaders before they can multiply and cause disease. For this reason, even during the COVID-19 pandemic, significant research has focused on nasal mucosal vaccines (1). These vaccines are not only effective but also help to relieve vaccine hesitancy and needle phobia. Additionally, their ease of use reduces the burden on medical staff during a pandemic.

Despite their advantages, most nasal vaccines currently under development utilize viral vector or attenuated virus platforms (2). While these viral vectored vaccines offer benefits, they also raise safety concerns, when administered intranasally, to immunocompromised patients. This route poses risks of neuroinflammation and other neurological adverse effects, as the vaccine can travel to the brain via the olfactory nerve. Beyond safety issues, a recent clinical trial revealed that a viral vector-based intranasal COVID-19 vaccine failed to generate adequate antigen-specific immunity (3), underscoring the need for an alternative intranasal vaccine strategy. There may be various reasons for this failure, but one potential solution to enhance immunogenicity is to diversify the vaccine platform. Among the available options, the subunit vaccine platform that employs adjuvant stands out as a proven and safe choice, commonly used in various intramuscular vaccines. This platform enables the design of vaccine antigens that align with the desired immune response, utilizing a limited number of epitopes, and generally results in fewer side effects (4). However, due to the inherently low immunogenicity of subunit antigens, the inclusion of an adjuvant is essential, particularly for intranasal vaccines. It is crucial to select an adjuvant capable of inducing mucosal immunity.

Recently, numerous studies have focused on adjuvants that stimulate the host’s innate immunity by utilizing pathogen-associated molecular patterns (PAMPs) to closely mimic the natural infection route of pathogens (5). In particular, research has examined TLR3 agonists like poly(I:C) as adjuvants for intranasal vaccines (6–8), TLR9 agonists such as CpG (9, 10), and a study investigating a nanoemulsion-based adjuvant (NE) that stimulates TLR2/4 in conjunction with RIG-I agonists (11). These studies consistently demonstrate protective efficacy against pathogens by inducing robust mucosal and systemic immunity in the host.

Previously, we developed a novel TLR3 agonist, Nexavant (NVT), which overcomes the limitations of existing poly(I:C) (12). By addressing the inherent heterogeneity of poly(I:C), we resolved issues that hindered the clinical application of existing poly(I:C), such as inconsistent responses, instability, and challenges in pharmacokinetic measurement (12–14). Mechanistically, both NVT and poly(I:C) activate TLR3. However, NVT activates TLR3 and RIG-I, but not MDA5, unlike poly(I:C) (14). Given that research indicates excessive immune responses can be induced by MDA5 (15, 16), NVT is expected to have a better safety profile than poly(I:C). Functionally, when formulated into a commercial influenza vaccine and administered to the lungs, NVT successfully induced resident memory CD4^+^ T cells in the lung mucosa, providing cross-protection against heterologous viruses and confirming its potential as an adjuvant for mucosal immunity (17). Moreover, when the CD4 epitope of tuberculosis was synthesized as a peptide, formulated with NVT, and administered into the lungs, it induced resident memory CD4^+^ T cells. This resulted in protective efficacy comparable to that of BCG, achieved with just two doses of the vaccine (18). Building on the results of these previous studies, this research aimed to evaluate the potential of using NVT as an adjuvant for intranasal vaccines, with a focus on immune responses, particularly antigen-specific IgA, an indicator of mucosal immunity, as well as systemic immune responses. When a commercially available inactivated split influenza vaccine was used as the antigen and NVT was formulated and administered intranasally, the results indicated that systemic humoral and cellular immune responses were induced, along with mucosal and cellular immune responses in the upper respiratory tract. Compared to existing intramuscular vaccines, we confirmed superior protective efficacy in a high-dose virus challenge model, attributed to the additional induction of mucosal immunity. Additionally, we observed that antigen-specific IgA was detected not only in the respiratory mucosa but also in other mucosal sites, including the oral cavity, vagina, and anus, following the intranasal vaccine. This finding confirms that the effect depended on type I interferon induced by NVT. Finally, to assess whether NVT could be applied to other platforms beyond proteins, we tested its efficacy with another vaccine antigen for a respiratory infectious disease related to meningitis. This meningitis antigen utilizes a platform that conjugates polysaccharides to carrier proteins. When NVT was formulated with this antigen and administered intranasally, it effectively induced both systemic and mucosal immunity. These research findings provide scientific evidence that NVT can serve as an adjuvant for nasal vaccines.

## 2 Materials and methods

### 2.1 Reagents and animals

The antibodies used in this study are listed in **Supplementary Table 1**. NVT was synthesized via *in vitro* transcription, as previously described (12). The monovalent H1N1 vaccine containing the A/Victoria/2570/2019 (IVR-215) strain from the 2022–2023 influenza season was provided by Il-Yang Pharmaceutical (Yongin, Korea). Specific pathogen-free, 6-week-old female C57BL/6 mice were purchased from Samtako Bio Korea (Kyounggi, Korea), while type I IFN receptor 1-deficient (IFNAR1^−/−^) mice on the C57BL/6 background were kindly provided by Professor Jae-Ho Cho (Chonnam National University Medical School, Korea). All animals were maintained under standard laboratory conditions with *ad libitum* access to food and water at the NAVI animal facility (Seoul, Korea).

### 2.2 Cell and virus

Madin-Darby canine kidney (MDCK) cells (CCL-34, ATCC) were cultured in Minimum Essential Eagle’s Medium (Welgene, Korea) supplemented with 10% heat-inactivated fetal bovine serum and 1% penicillin-streptomycin solution. The BALB/c mouse-adapted influenza A/Korea/2785/2009 (H1N1) strains, obtained from the Korea Disease Control and Prevention Agency (Cheongju, Chungcheongbuk-do, Korea), were propagated in MDCK cells and subsequently quantified using a plaque assay. The 50% lethal dose (LD_50_) was determined by infecting mice with serial dilutions of the virus.

### 2.3 Polysaccharides and carrier proteins

*Neisseria meningitidis* serogroup C (ATCC 13102) was used in this study. The bacterial strain was cultured in a chemically defined medium under controlled bioreactor conditions to optimize polysaccharide production. After centrifugation to remove bacterial cells, impurities including proteins, nucleic acids, and endotoxins were precipitated from the supernatant by the addition of salt and ethanol. Diafiltration was applied throughout the purification process to remove low-molecular-weight substances, such as media components and salts, while exchanging the solvent with water. The carrier protein, CRM197, was expressed in *Escherichia coli* BL21(DE3) carrying a plasmid encoding the CRM197 protein. The protein was extracted from cultured cells by osmotic shock and purified through two sequential chromatography steps: anion exchange and ceramic hydroxyapatite chromatography. During the final chromatography step, the buffer was exchanged for phosphate-buffered saline (PBS) containing 0.005% polysorbate 80.

### 2.4 Preparation of meningococcal conjugate vaccine

MCV was synthesized using purified polysaccharide and CRM197. First, the carrier protein was derivatized with an adipic acid dihydrazide (ADH) linker using N-(3-dimethylaminopropyl)-N’-ethylcarbodiimide (EDAC) as a coupling agent. The resulting ADH-linked CRM197 was purified by diafiltration against sodium phosphate buffer to remove excess reactants. Subsequently, the hydroxyl groups of the polysaccharide were activated with 1-cyano-4-dimethylaminopyridinium tetrafluoroborate (CDAP) for 5 min at room temperature under alkaline conditions. This activated intermediate was then conjugated to the ADH-linked CRM197 by overnight incubation at room temperature. The final conjugate was purified by diafiltration against PBS to remove residual reactants and formulated in PBS containing 0.005% polysorbate 80.

### 2.5 Collection of mucosal samples

To analyze mucosal antibody responses, various samples—including nasal wash, vaginal wash, saliva, and feces—were collected. Nasal washes were collected by inserting a catheter into the nasal cavity and flushing with 500 μl of PBS using a syringe. Vaginal washes were obtained by gently washing the vaginal lumen with 100 μl of PBS. Saliva was collected by aspiration using a micropipette following intraperitoneal administration of pilocarpine hydrochloride (Sigma-Aldrich) at 2 mg/kg body weight to stimulate secretion. Fecal pellets were collected directly from mice and suspended in PBS at a concentration of 100 mg/ml, followed by vortexing and centrifugation to obtain supernatants for antibody assays. All samples were stored at −80 °C until analysis.

### 2.6 Single-cell preparation

Nasal-associated lymphoid tissue (NALT) was isolated from euthanized mice by carefully excising the upper incisors and surrounding soft tissues to expose the nasal cavity. The palate containing bilateral NALT aggregates adjacent to the nasal septum was detached using fine forceps. Excised tissues were placed in PBS supplemented with 1% FBS and mechanically dissociated by gently pressing with the plunger of a 1 mL syringe. The resulting suspension was filtered through a 70 μm cell strainer to obtain a single-cell suspension. Spleens were aseptically harvested and mechanically dissociated by pressing through a 70 μm cell strainer using the plunger of a syringe. Following red blood cell lysis with 1× RBC lysis buffer (BioLegend, San Diego, CA, USA) for 4–5 min on ice, the cells were washed with PBS and resuspended in complete RPMI-1640 medium (RPMI-1640 supplemented with 10% FBS, 1% penicillin-streptomycin, and 2 mM L-glutamine) to obtain a single-cell suspension.

### 2.7 RNA extraction and quantitative real-time PCR

Total RNA was extracted from the NALT of C57BL/6 mice using TRIzol reagent (Thermo Fisher Scientific) according to the manufacturer’s instructions. RNA concentration and purity were assessed using a NanoDrop spectrophotometer (Molecular Devices, San Jose, CA, USA). Samples with absorbance ratios of 260/280 between 1.8 and 2.0 and 260/230 between 2.0 and 2.2 were considered to be of high purity. Quantitative real-time PCR was performed using the One Step TB Green PrimeScript RT-PCR Kit (Takara Bio, Shiga, Japan) following the manufacturer’s protocol. Briefly, RNA samples were combined with 2× One Step TB Green RT-PCR Buffer 4, PrimeScript 1 Step Enzyme Mix 2, forward and reverse primers (10 μM), and RNase-free water in a 96-well reaction plate. The reactions were run on a CFX Real-Time PCR System (Bio-Rad, Hercules, CA, USA) with the following thermal profile: reverse transcription at 42°C for 5 min, followed by 40 cycles of 95°C for 10 s and 55°C for 30 s. Gene expression levels were normalized to the reference gene HPRT and analyzed using the 2^−ΔΔCt^ method. Primers were synthesized by Cosmogenetech (Seoul, Korea), and the sequences are listed in **Supplementary Table 2**.

### 2.8 Analysis of innate immune response

C57BL/6 mice were intranasally administered 1 μg of NVT in a total volume of 5 μl. Twenty-four hours post-administration, NALT cells were stained with a fixable viability dye to exclude dead cells and then labeled with anti-mouse CD11b, F4/80, Ly6G, CD11c, and MHC-II antibodies. Macrophages (CD11b^+^F4/80^+^), neutrophils (CD11b^+^Ly6G^+^), and dendritic cells (MHC-II^+^CD11c^+^) in NALT were quantified by flow cytometry (**Supplementary Figure 1**). To assess dendritic cell activation, dendritic cells were gated and analyzed for surface expression of CD40, CD80, CD86, and MHC-II using a NovoCyte flow cytometer (ACEA Biosciences, San Diego, CA, USA). IFN-β levels in NALT lysates were measured by sandwich ELISA according to the manufacturer’s protocol (BioLegend, San Diego, CA, USA).

### 2.9 Immunization and viral challenge

To compare the effects of intramuscular and intranasal administration routes and the adjuvant NVT, C57BL/6 mice were immunized twice at 3-week intervals with 1 μg of H1N1 (A/Victoria/2570/2019 (IVR-215)) antigen alone or formulated with 10 μg of NVT (H1N1+NVT) via intramuscular injection (50 μl) or intranasal administration (5 μl). Three weeks after the final immunization, hemagglutination inhibition (HAI) titers were measured in serum and nasal wash samples. H1N1-specific antibody responses were assessed by Enzyme-Linked Immunosorbent Assay (ELISA) in serum, nasal wash, vaginal wash, saliva, and feces, and H1N1-specific T cell responses were analyzed in NALT and spleen. At this same time point, mice were challenged intranasally with either 100 LD_50_ or 5,000 LD_50_ of the H1N1 virus (A/Korea/2785/2009) in a volume of 50 μl. For mechanistic studies, wild-type (WT) and IFNAR1^-^/^-^ mice were intranasally immunized twice at 3-week intervals with H1N1+NVT (5 μl). Immune responses were evaluated three weeks after the final immunization as described above, and mice were challenged intranasally with 100 LD_50_ of the H1N1 virus (A/Korea/2785/2009) in a volume of 50 μl at the same time point. For the meningitis vaccine study, C57BL/6 mice were immunized twice at 3-week intervals with 1 μg of MCV alone via intramuscular injection (50 μl) or intranasal administration (5 μl), or with MCV formulated with 10 μg of NVT (MCV+NVT) via intranasal administration (5 μl). Three weeks after the final immunization, meningococcal serogroup C polysaccharide (MenC-PS)-specific antibody levels and serum bactericidal activity (SBA) titers were measured in serum and nasal wash samples.

### 2.10 Antigen-specific antibody responses

For H1N1-specific antibody detection, 96-well microplates were coated with 5 μg/ml of inactivated H1N1 (A/Victoria/2570/2019 (IVR-215)) antigen overnight at 4°C. For MenC-PS-specific antibody detection, plates were pre-coated with 3 μg/ml of poly-lysine at 37°C for 30 min, followed by coating with 10 μg/ml of MenC-PS antigen overnight at 4°C. For standard curve construction, separate wells were coated with 2 μg/ml of goat anti-mouse IgG antibody overnight at 4°C. All plates were blocked with 1% skim milk to prevent non-specific binding. Serially diluted mouse IgG antibodies for the standard curve and samples were incubated for 2 h at room temperature. Serum samples and mouse IgG standards were incubated with horseradish peroxidase (HRP)-conjugated anti-mouse IgG antibodies, whereas nasal wash, vaginal wash, saliva, and feces samples were incubated with HRP-conjugated anti-mouse IgA antibodies. Plates were developed using 3,3′,5,5′-tetramethylbenzidine substrate, and the reaction was stopped with 0.5 M HCl. Absorbance was measured at 450–590 nm using a Multiskan Sky microplate reader (Thermo Fisher Scientific). Serum antibody titers were calculated as arbitrary units relative to the standard curve, while mucosal antibody levels were represented as optical density (OD) values at 450–590 nm.

### 2.11 Antigen-specific T cell responses

Spleens and NALT were harvested from vaccinated mice and dissociated into single-cell suspensions. To evaluate H1N1-specific T cell responses, the cells were stimulated with 5 μg/ml H1N1 (A/Victoria/2570/2019 (IVR-215)) antigen overnight at 37°C. GolgiPlug (BD Biosciences) was then added to block cytokine secretion, and the cells were incubated for an additional 4–6 h. Fc receptors were blocked using anti-mouse CD16/CD32 antibodies, followed by viability staining with a fixable live/dead dye. Surface staining was performed using fluorochrome-conjugated antibodies against CD4, CD8α, and CD44. Cells were subsequently fixed and permeabilized with Cytofix/Cytoperm solution (BD Biosciences) and stained intracellularly with anti-mouse IFN-γ antibodies. After washing, cells were resuspended in FACS buffer (PBS supplemented with 1% FBS and 0.1% sodium azide). Antigen-specific CD4^+^ and CD8^+^ T cells producing IFN-γ (CD44^+^IFN-γ^+^) in spleen and NALT were quantified by flow cytometry (**Supplementary Figure 2**).

### 2.12 Hemagglutination inhibition assay

Serum and nasal wash samples were mixed with receptor-destroying enzyme (RDE) and PBS at a ratio of 1:3:6 and incubated overnight at 37 °C to remove nonspecific interference. After RDE treatment, serum samples were heat-inactivated at 56 °C for 30 min. Samples were then serially diluted two-fold in 96-well V-bottom plates. Each dilution was incubated with 4 hemagglutination units of H1N1 (A/Victoria/2570/2019 (IVR-215)) antigen at room temperature for 30 min, followed by the addition of 1% chicken red blood cells and a further 30 min incubation. The HAI titer was determined as the highest dilution that completely prevented red blood cell agglutination. A titer of 1:40 or greater was considered protective against influenza infection.

### 2.13 Serum bactericidal assay

Serum bactericidal activity against *Neisseria meningitidis* serogroup C was assessed using heat-inactivated mouse serum and baby rabbit complement (BRC; MP Biomedicals), following a standardized protocol. Mouse sera were heat-inactivated at 56°C for 30 min to eliminate endogenous complement activity. Serum samples were serially diluted two-fold in 96-well plates using assay buffer (Dulbecco’s phosphate-buffered saline supplemented with 0.1% glucose), with 50 μl of each dilution added per well. An equal volume (50 μl) of a suspension containing mid-log-phase *N. meningitidis* serogroup C and 10% (v/v) BRC was then added to each well, resulting in a final reaction volume of 100 μl and a final BRC concentration of 5%. Control wells containing bacteria and complement, without serum, were included to measure non-specific killing. After incubation for 1 h at 37°C, 10 μl from each well was spotted onto pre-dried soy agar plates and allowed to air-dry. The plates were subsequently overlaid with top agar (soy medium containing 1% agar and 100 μg/ml 2,3,5-triphenyltetrazolium chloride) and incubated overnight at 37°C. Bacterial colonies were enumerated the following day. SBA titers were defined as the reciprocal of the highest serum dilution resulting in ≥ 50% reduction in colony counts relative to the control wells. A titer ≥ 8 was considered indicative of protective bactericidal activity.

### 2.14 Statistical analysis

All data analyses were performed using GraphPad Prism version 10.4.0 for Windows (GraphPad Software, La Jolla, CA, USA; www.graphpad.com). Statistical comparisons between two groups were conducted using the nonparametric Mann–Whitney U test. For multiple group comparisons, one-way analysis of variance (ANOVA) followed by Tukey’s *post hoc* test was applied. Statistical significance was defined as follows: *p < 0.05, **p < 0.01, ***p < 0.001, and ****p < 0.0001.

## 3 Result

### 3.1 Intranasal immunization with NVT enhances the expression of viral nucleic acid sensors and activates innate immune cells in a manner dependent on type I interferon

**Figure 1:**
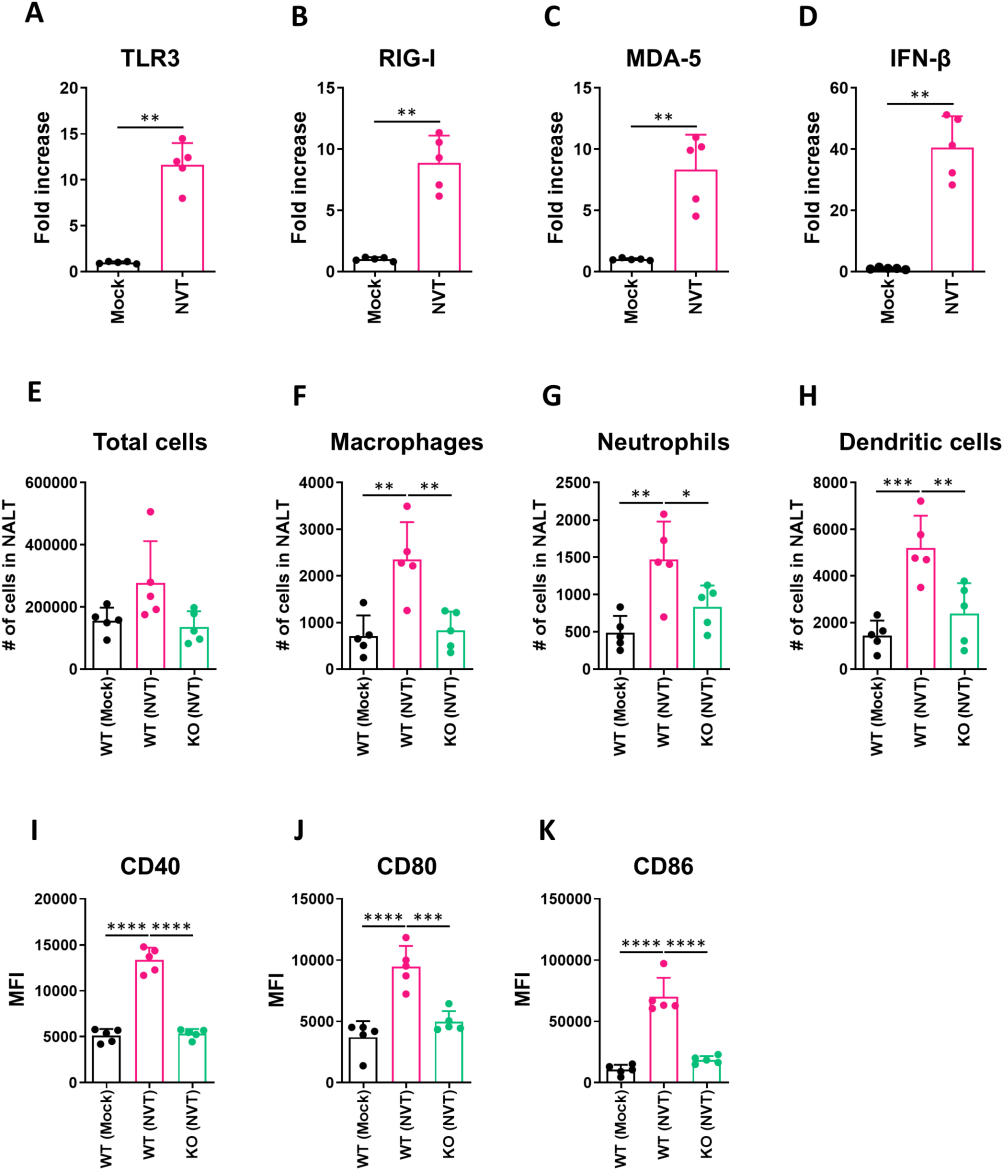
Expression of viral nuclear sensor and innate immune responses in NALT. **(A-D)** C57BL/6 mice (n = 5 per group) were intranasally administered 1 μg of NVT. NALT was collected 5 h later for RT-qPCR. mRNA levels of TLR3 **(A)**, RIG-I **(B)**, MDA5 **(C)**, and IFN-β **(D)** were normalized to HPRT1 and expressed relative to mock controls. **(E-K)** Wild-type (WT) and IFNAR1^-^/^-^ mice (n = 5 per group) received the same treatment, and NALT was collected 24 h later for flow cytometry. Total NALT cells **(E)**, macrophages (CD11b^+^F4/80^+^) **(F)**, neutrophils (CD11b^+^Ly6G^+^) **(G)**, and dendritic cells (MHC-II^+^CD11c^+^) **(H)** were quantified. Absolute numbers were calculated by multiplying total cells by frequency. Mean fluorescence intensity (MFI) of CD40 **(I)**, CD80 **(J)**, and CD86 **(K)** on CD11c^+^MHC-II^+^ dendritic cells was measured. Data are presented as mean ± SD. These experiments were independently performed twice. Statistical analysis: **A-D**, two-sided Mann-Whitney U test; **E–K**, one-way ANOVA with Tukey’s multiple comparisons test. *P < 0.05; **P < 0.01; ***P < 0.001; ****P < 0.0001.

When pathogens such as viruses invade, the mucosa functions not only as a physical barrier but also as a detection system. Epithelial cells identify these invaders through pattern recognition receptors (PRRs), including TLR, RIG-I, and MDA5. This recognition triggers the activation of the innate immune system, resulting in the secretion of interferon. An effective vaccine aims to closely mimic the natural immune response to a pathogen. Thus, we aimed to determine the changes in the mucosal immune system *in vivo* following intranasal administration of NVT. Previous studies have indicated that NVT increases the expression levels of viral nucleic acid sensors in draining lymph nodes, which in turn elevate interferon beta when administered intramuscularly (12). In this study, we measured the expression levels of viral nucleic acid sensors and interferon beta after administering NVT intranasally. Five hours post-administration, we observed a significant increase in the expression levels of TLR3, RIG-I, and MDA5 in the nasal-associated lymphoid tissue (NALT) (**Figures 1A-C**), along with a notable increase in interferon beta (**Figure 1D**). Previous experiments with reporter cell lines have shown that NVT does not directly activate MDA5 (14). However, our results indicate that MDA5 was activated, likely due to the paracrine effect of type I interferon secreted by NVT, which indirectly activates MDA5 in surrounding cells, as demonstrated in other research (19).

After confirming that intranasally administered NVT successfully activated PRRs in the NALT, we examined the changes in the innate immune response induced in the host’s NALT. Specifically, we compared the responses in IFNAR1^-/-^ (KO) mice to explore the role of the key effector molecule interferon beta, which is induced by NVT. In the group that received NVT intranasally, the total number of NALT cells showed a slight increase compared to the mock group; however, this difference was not statistically significant (**Figure 1E**). Notably, the populations of macrophages, neutrophils, and dendritic cells in the NALT significantly increased. However, the increase in immune cells in the NALT following NVT administration was not observed in the KO mice (**Figures 1F–H**).

We also assessed the activity of dendritic cells, which play a crucial role in activating adaptive immunity within the NALT. In the wild-type (WT) group receiving intranasal NVT, we confirmed a significant increase in the activation markers CD40, CD80, and CD86 on dendritic cells (**Figures 1I–K**). Conversely, dendritic cells in the KO mice did not exhibit activation even after NVT administration. In summary, nasal administration of NVT activates the innate immunity of the NALT, and this activation is dependent on type I interferon.

### 3.2 Comparison of the immunological profiles and protective efficacy of commercially available influenza vaccines administered intramuscularly and intranasally with NVT adjuvant

**Figure 2:**
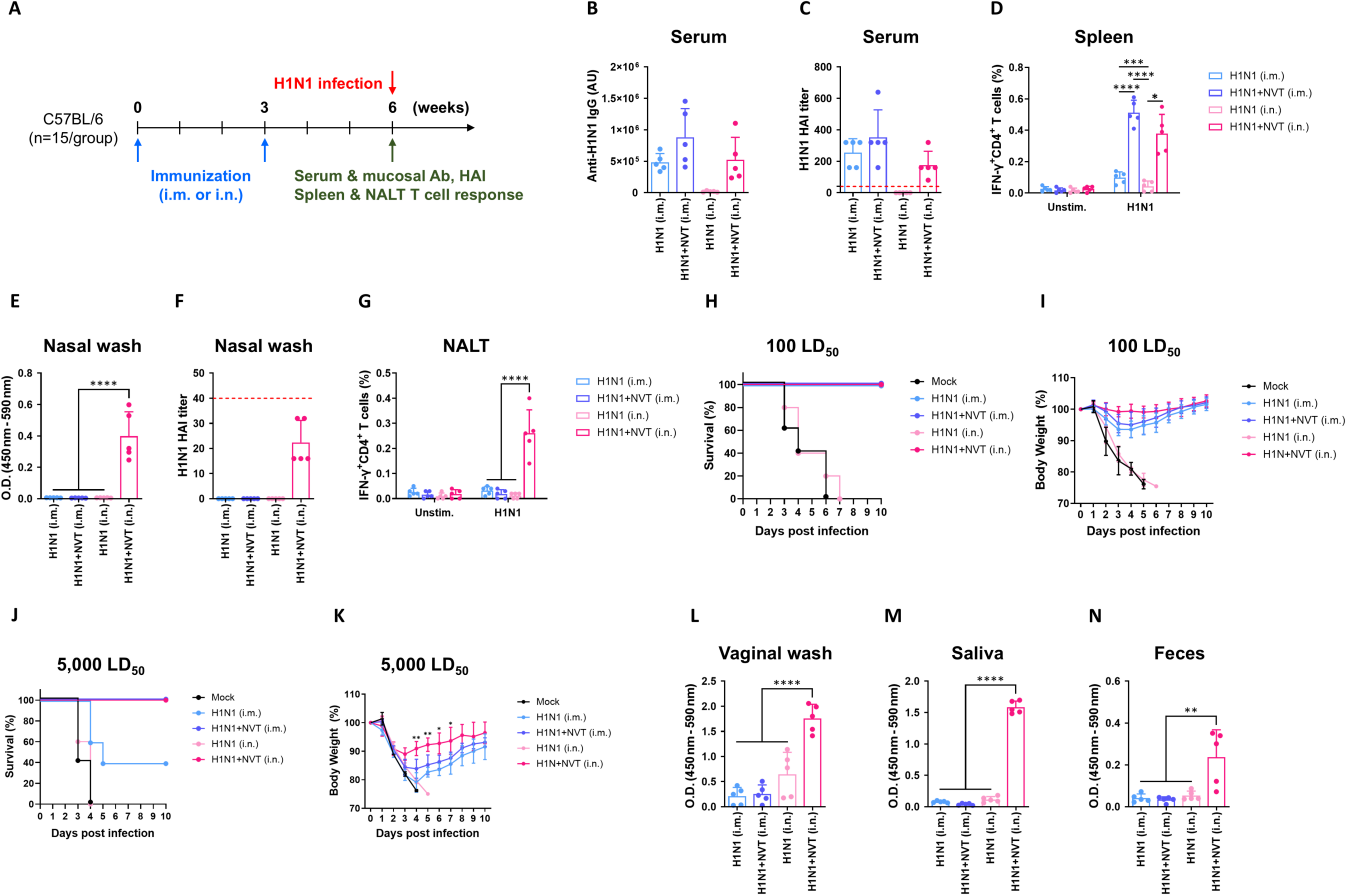
Immunological comparison between NVT-adjuvanted intranasal influenza vaccine and commercial influenza vaccine. **(A)** Experimental design. C57BL/6 mice (n = 15 per group) were immunized twice at 3-week intervals either intramuscularly (i.m.) or intranasally (i.n.) with 1 µg of H1N1, alone or formulated with 10 µg of NVT (H1N1+NVT), resulting in four groups: H1N1 (i.m.), H1N1+NVT (i.m.), H1N1 (i.n.), and H1N1+NVT (i.n.). Three weeks after the final immunization, five mice per group were used for immune response analysis, and the remaining ten mice were i.n. challenged with either 100 or 5,000 LD_50_ of H1N1 virus (A/Korea/2785/2009) (n = 5 per challenge dose). **(B, C)** H1N1-specific serum IgG **(B)** and HAI titers **(C)**. **(D)** H1N1-specific IFN-γ^+^CD4^+^ T cell response in the spleen. **(E, F)** H1N1-specific IgA in nasal washes **(E)** and HAI titers **(F)**. An HAI antibody titer of 1:40 (red dotted line) was considered protective against influenza infection. **(G)** IFN-γ^+^CD4^+^ T cell response in NALT. **(H, I)** Survival **(H)** and body weight **(I)** after intranasal infection with 100 LD_50_ of H1N1 (n = 5 per group). **(J, K)** Survival **(J)** and body weight **(K)** after intranasal infection with 5,000 LD_50_ of H1N1 (n = 5 per group). Survival data are presented as Kaplan–Meier survival curves. Statistical significance in body weight differences between the H1N1+NVT (i.m.) and H1N1+NVT (i.n.) groups was analyzed using a two-sided Mann–Whitney U test. **(L-N)** H1N1-specific IgA levels in vaginal washes **(L)**, saliva **(M)**, and feces **(N)** measured by antigen-specific ELISA. Data are presented as mean ± SD. These experiments were independently performed at least three times. Statistical analyses between groups were performed using one-way ANOVA with Tukey’s multiple comparisons test. *P < 0.05; **P < 0.01; ***P < 0.001; ****P < 0.0001.

Having established that intranasally administered NVT successfully induced innate immunity in the NALT, we sought to determine whether this innate immune activation also induced adaptive immunity. To do this, we compared the systemic and mucosal immune profiles of commercially available inactivated split influenza H1N1 antigens, both with and without NVT as an adjuvant, and administered either intramuscularly or intranasally. As indicators of systemic immunity, we measured antigen-specific IgG in serum and the antigen-specific T cell response in the spleen. For serum, functional antibody titers were determined using a hemagglutination inhibition (HAI) assay. For mucosal immunity, we measured antigen-specific IgA in nasal wash and the antigen-specific T cell response in the NALT, with functional antibody titers in the nasal wash also assessed via HAI assay. The experimental schedule involved administering the vaccine for each condition twice at three-week intervals, followed by an immunoassay three weeks after the last administration (**Figure 2A**).

In terms of serum antigen-specific IgG, which reflects humoral immune responses within systemic immunity, responses were observed in all intramuscularly immunized groups. Although the H1N1+NVT (i.m.) group showed slightly higher IgG levels compared to H1N1 alone, this difference did not reach statistical significance (**Figure 2B**). Similarly, HAI titers showed a comparable pattern, with no statistically significant increase in the H1N1+NVT (i.m.) group (**Figure 2C**). No antigen-specific IgG was detected in the group receiving H1N1 intranasally without NVT, whereas the intranasal H1N1+NVT group showed IgG levels similar to those observed in the intramuscular groups. In contrast, antigen-specific CD4^+^ T cell responses, indicative of systemic cellular immunity, were significantly enhanced compared to the H1N1 alone groups in all NVT-containing groups regardless of the administration route (**Figure 2D**), supporting the capacity of NVT to augment systemic cellular immune responses under both intramuscular and intranasal immunization conditions. Consistent with a previous study (17), no antigen-specific CD8^+^ T cell response was observed with the addition of NVT to this antigen.

In the nasal wash, which serves as an indicator of mucosal immunity induction, antigen-specific IgA was detected only in the group administered H1N1+NVT intranasally, whereas no antigen-specific IgA was observed when NVT was used via the intramuscular route (**Figure 2E**). Notably, a slight HAI titer was detectable in the nasal wash of the intranasally administered H1N1+NVT group, albeit weak, suggesting a potential contribution to viral neutralization (**Figure 2F**). These results indicate that, despite the modest HAI activity, intranasal NVT-adjuvanted vaccination effectively induces antigen-specific mucosal antibody responses. In contrast to the spleen, antigen-specific CD4^+^ T cell responses in the NALT were observed solely in the group receiving intranasal H1N1+NVT, indicating a difference from the systemic cellular immune response pattern (**Figure 2G**).

To determine whether these immunological differences resulted in functional outcomes, mice were challenged with the influenza virus. In both the group that received H1N1 alone intranasally, which did not develop systemic or mucosal immune responses, and the mock control group, all mice perished after being challenged with an intermediate dose (100 LD_50_). In contrast, all mice in the remaining immunized groups survived (**Figure 2H**). Mice immunized via the intramuscular route exhibited a transient loss of body weight followed by full recovery, whereas mice receiving intranasal H1N1+NVT showed minimal to no body weight loss throughout the observation period (**Figure 2I**). When challenged with a high dose (5,000 LD_50_), approximately 60% of the mice immunized intramuscularly with H1N1 alone succumbed to infection, whereas all mice in the NVT-adjuvanted groups survived regardless of the administration route (**Figure 2J**). Importantly, although all surviving mice experienced some weight loss, the reduction was significantly less in the intranasally administered H1N1+NVT group compared to the intramuscular group (**Figure 2K**), indicating that mucosal immunity induced by intranasal vaccination provided superior protection. In addition to protecting against influenza, mucosal immunity is known to influence other mucosal tissues in the body through cross-communication (20). We hypothesized that immunity could be induced in mucosa other than the respiratory tract when the NVT-adjuvanted vaccine was administered intranasally. To investigate this, we measured antigen-specific IgA in saliva, vaginal wash, and feces, representing the oral, vaginal, and anal mucosa, respectively. Significant amounts of antigen-specific IgA were detected in saliva, vaginal wash, and feces compared to the control (**Figures 2L-N**). In summary, we confirmed that the combination of NVT and intranasal administration is essential for inducing mucosal immunity, and that crosstalk occurs with other mucosal tissues.

### 3.3 Systemic and mucosal immunity induced by intranasal influenza vaccines adjuvanted with NVT occurs in a type I interferon-dependent manner

**Figure 3:**
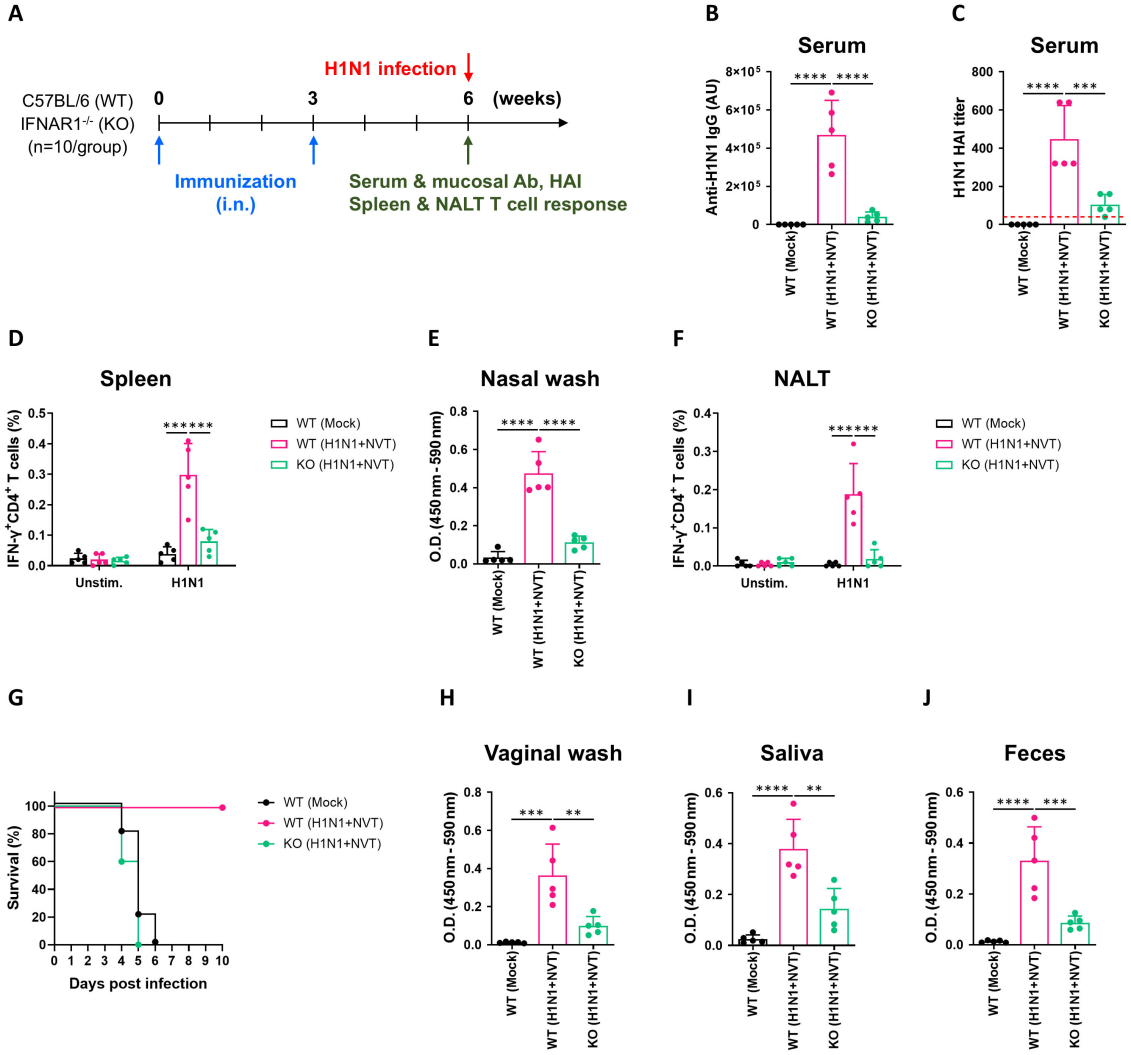
NVT-adjuvanted intranasal influenza vaccines induce mucosal and systemic immunity in a type I interferon-dependent manner. **(A)** Experimental design. WT (n = 10 per group) and IFNAR1^-^/^-^ mice (n = 10 per group) were immunized i.n. with H1N1+NVT twice at three-week intervals. Three weeks after the final immunization, immune responses were analyzed (n = 5 per group), or mice were intranasally challenged with 100 LD_50_ of H1N1 virus (A/Korea/2785/2009) (n = 5 per group). **(B, C)** H1N1-specific serum IgG **(B)** and HAI titers **(C)**. An HAI antibody titer of 1:40 (red dotted line) was considered protective against influenza infection. **(D)** H1N1-specific IFN-γ^+^CD4^+^ T cell response in the spleen. **(E)** H1N1-specific IgA in nasal washes. **(F)** IFN-γ^+^CD4^+^ T cell response in NALT. **(G)** Survival following intranasal infection with 100 LD_50_ of H1N1 (n = 5 per group). Survival data are presented as Kaplan–Meier survival curves. **(H-J)** H1N1-specific IgA levels in vaginal washes **(H)**, saliva **(I)**, and feces **(J)** measured by antigen-specific ELISA. Data are presented as mean ± SD. These experiments were performed once. Statistical analyses between groups were performed using one-way ANOVA with Tukey’s multiple comparisons test. **P < 0.01; ***P < 0.001; ****P < 0.0001.

After confirming that systemic and mucosal immunity were effectively induced when NVT was used as an adjuvant for intranasal vaccination, we conducted an experiment to further analyze these immune responses. We intranasally administered the H1N1+NVT vaccine to type I interferon knockout (KO) mice to determine whether the activity of type I interferon-dependent innate immunity observed in NALT is linked to adaptive immunity (**Figure 3A**). As seen in the previous experiment, antigen-specific IgG was robustly induced in the group of WT mice receiving the H1N1+NVT vaccine, while it was barely detectable in the KO mice (**Figure 3B**). HAI results in serum showed a similar pattern to that of antigen-specific IgG (**Figure 3C**). The systemic antigen-specific CD4^+^ T cell response, induced in WT mice by nasal vaccination, was not observed in the KO mice (**Figure 3D**). Furthermore, neither antigen-specific IgA in the nasal wash nor the antigen-specific CD4^+^ T cell response in the NALT was induced (**Figures 3E, F**), and all mice succumbed to the intermediate dose of influenza virus challenge (**Figure 3G**). Antigen-specific IgA, which was effectively induced in the respiratory mucosa as well as other mucosal sites, was completely absent in the KO mice (**Figures 3H-J**). Taken together, both systemic and mucosal immunity were compromised in KO mice, leading to the conclusion that the immune responses induced by the intranasal administration of H1N1+NVT are dependent on type I interferon.

### 3.4 NVT administered intranasally with meningitis polysaccharide-carrier protein conjugate antigen induces mucosal and systemic immunity

Having elucidated the overall immune response and its mechanism when NVT was applied to an influenza vaccine and administered intranasally, we questioned whether systemic and mucosal immunity could be induced when NVT was used as an adjuvant for other antigens administered intranasally. To investigate this, we selected an antigen from a meningitis vaccine. Meningitis was chosen because a commercial product has been released, and it is a disease where droplet transmission is a natural route of infection, making the application of an intranasal vaccine advantageous. Furthermore, since we have already confirmed that mucosal immunity to protein antigens is induced through influenza antigens, we aimed to verify whether mucosal immunity could also be induced by polysaccharides, which are a different modality.

**Figure 4:**
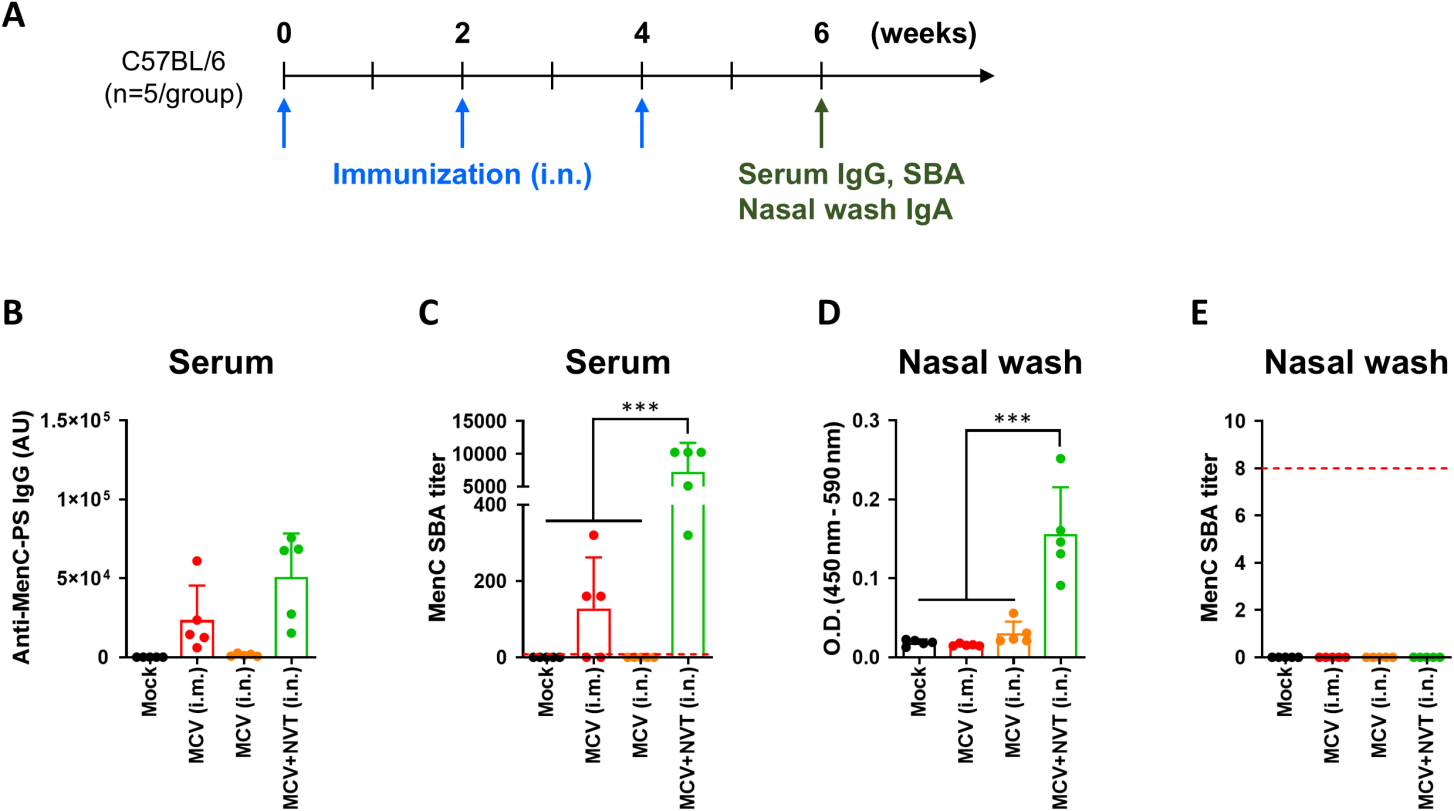
The application of NVT to the meningitis polysaccharide conjugate vaccine platform demonstrates improved efficacy, even when administered intranasally. **(A)** Experimental design. C57BL/6 mice (n = 5 per group) were immunized three times at 2-week intervals either i.m. or i.n. with 1 µg of meningococcal conjugate vaccine (MCV), alone or formulated with 10 µg of NVT—resulting in three groups: MCV (i.m.), MCV (i.n.), and MCV+NVT (i.n.). Serum and nasal wash samples were collected two weeks after the final immunization. **(B)** Meningococcal serogroup C polysaccharide (MenC-PS)-specific serum IgG titers. **(C)** Serum bactericidal antibody (SBA) titers against MenC. **(D)** MenC-PS-specific IgA levels in nasal wash. **(E)** SBA titers against MenC measured in nasal washes. Data are presented as mean ± SD. These experiments were performed once. Statistical analyses between groups were performed using one-way ANOVA with Tukey’s multiple comparisons test. ***P < 0.001.

To confirm this, we compared the systemic and mucosal immune profiles of the meningococcal conjugate vaccine (MCV) serotype C administered intramuscularly, intranasally, and intranasally with NVT as an adjuvant. Since the epitope of the meningococcal vaccine is a polysaccharide, we did not analyze cellular immunity. The experimental schedule involved three administrations over two weeks for each condition, with various immune analyses performed two weeks after the final administration (**Figure 4A**). As a result, an appropriate amount of antigen-specific IgG was detected in the group administered MCV intramuscularly, but almost none was found in the group administered it intranasally (**Figure 4B**). A slightly greater amount of antigen-specific IgG was detected in the group administered MCV+NVT intranasally compared to the group that received MCV intramuscularly, though this difference was not statistically significant (**Figure 4B**). To evaluate the production of functional antibodies in the serum, an SBA was performed, yielding results that corresponded to the antigen-specific IgG values (**Figure 4C**). Notably, in the group that received only MCV intranasally, no SBA titer was observed, while in the group that received MCV+NVT intranasally, a significantly higher SBA titer was noted compared to the group that received only MCV intramuscularly.

To confirm mucosal immunity, we measured antigen-specific IgA in nasal wash, and, similar to the findings with influenza, only the group administered MCV+NVT showed a significant detection value (**Figure 4D**). We also performed the SBA using nasal wash; however, no SBA titer was detected in any group in the nasal wash (**Figure 4E**). Since it is generally understood that IgA does not activate the classical pathway of complement (21), we concluded that it is not possible to confirm the function of IgA using the SBA. Nevertheless, the detection of antigen-specific IgA suggests that mucosal immunity was induced. In summary, when NVT was used as an adjuvant for nasal vaccination, systemic and mucosal immunity were successfully induced even with a polysaccharide-protein conjugate vaccine.

## 4 Discussion

Intranasal vaccines can offer improved protective efficacy by inducing immunity in the nasal mucosa, the primary site of infection for respiratory pathogens. In this study, we conducted a proof-of-concept investigation to evaluate the use of NVT, a TLR3 agonist, as an immune adjuvant for nasal vaccines. To enhance clinical relevance, we tested the vaccines using existing commercially available platforms as antigens, including the inactivated split vaccine platform and the polysaccharide-protein conjugate vaccine platform. Given that droplet transmission is the main route of infection for both diseases, we considered this an ideal model to demonstrate the potential of an intranasal vaccine. Our results confirmed that both intranasal vaccines, utilizing the two platforms as antigens, successfully induced systemic and mucosal immunity. We also experimentally demonstrated that the mucosal vaccine exhibited superior efficacy compared to the intramuscular vaccine, particularly against influenza, using a high-dose challenge model. Beyond efficacy, we established a safety advantage; a preclinical GLP toxicity study on NVT showed that intranasal administration was better tolerated than intramuscular administration (**Supplementary Table 3**).

In this study, we strictly controlled the volume of the nasal vaccine to less than 10 µl. Previous research has confirmed that when the nasal administration volume in mice exceeds 10 µl, the vaccine can enter the lungs (17). While there is no difference in the effect on the upper respiratory tract—since the vaccine administered intranasally passes through it—the implications of whether it reaches the lower respiratory tract and enters the lungs are significant. First, the development of nasal and lung-targeting vaccines involves distinct considerations. For instance, the delivery method differs greatly; one may use a nasal sprayer, which is generally easier to use, or a device like a nebulizer. Additionally, toxicity tests must be conducted with the understanding that delivery may occur in the lungs, necessitating careful consideration from the preclinical stage onward. Second, and perhaps more importantly for researchers validating the concept, whether the vaccine reaches the lungs affects the strength of the systemic immune response. In our previous study, we found that lung-resident memory CD4^+^ T cells formed only when the intranasally administered vaccine reached the lungs (17). At that time, we focused solely on lung CD4^+^ T_RM_ and did not analyze other immunological factors influenced by the administered volume. In this study, having developed an adjuvant that targets the nasal cavity rather than the lungs, we reanalyzed this issue and confirmed that when delivered to the lungs, both serum antigen-specific IgG levels—indicative of a systemic immune response—and the antigen-specific T cell response in the spleen increased. However, there was no difference in the antigen-specific IgA levels observed in the mucosa (**Supplementary Figure 3**). Thus, the administration volume is crucial in studying intranasal vaccines; if the volume exceeds 10 µl, the vaccine may enter the lungs, potentially exaggerating the systemic immune response.

We confirmed that antigen-specific IgA was detected not only in the nasal mucosa but also in other mucosal tissues, such as the oral cavity, colon, and vagina. This evidence suggests that immune crosstalk between different mucosal tissues in the body can be induced by this vaccine platform. This finding aligns with previous reports indicating that respiratory pathogen infections also affect the intestinal mucosa (20, 22, 23). Such crosstalk is plausible, considering that the primary functions of TLR3 and RIG-I, the main effector receptors of NVT, are to recognize viral components and activate the immune response. Chemokines play a significant role in facilitating immune crosstalk between mucosal tissues (24). In our previous study, we analyzed the chemokine profile induced by NVT when delivered to the lungs, which can be regarded as the respiratory mucosa, and confirmed that CCL2, CCL5, and the CXCR3/ligand axis were predominantly induced (17). The involvement of these cytokines in mucosal immunity has been partially elucidated through several studies (25–28). Notably, a recent study demonstrated that when mRNA vaccines are administered intranasally, they promote differentiation into plasma cells that secrete IgA via signaling through the CXCR3 axis (29). However, the chemokine profile results mentioned above pertain specifically to NVT delivered to the lungs, and since the study did not screen all chemokines, additional research is necessary. Future investigations should focus on these chemokines and their effects on immune cells in mucosal tissues, as well as the resulting immune responses. If these characteristics are well identified, they could provide a logical basis for developing vaccines for sexually transmitted diseases or infectious diseases transmitted through the mouth as intranasal vaccines.

The data presented in this study have the limitation that their efficacy was tested only in mice. This study was conducted in mice during the early stages of concept verification, but the structure of the nasal cavity varies across species. Notably, significant differences exist between mice and humans in the structure of the turbinates, which directly affect vaccine contact during intranasal administration (30). Therefore, additional research is necessary to determine whether NVT is effective in larger animals or primates when administered intranasally. In conclusion, this study demonstrated that NVT, a novel TLR3 agonist that addresses the shortcomings of existing poly(I:C), can induce both mucosal and systemic immunity when subunit antigens are administered intranasally to mice using an adjuvant. Specifically, it has shown potential for inducing mucosal immunity against polysaccharide-protein conjugate antigens that are challenging to access with the viral vector platform, the most widely used existing mucosal vaccine platform, or the mRNA vaccine platform, which is currently gaining significant attention. This provides scientific evidence for broader applications. We believe that this approach can serve as a viable option for other intranasal vaccines currently under development or planned for future development.

## Data Availability

The original contributions presented in the study are included in the article/**Supplementary Material**. Further inquiries can be directed to the corresponding authors.
