# Supplementary material for: Intranasal vaccines adjuvanted with Nexavant demonstrate robust protective efficacy by inducing both mucosal and systemic immunity in a murine model
Source: Front Immunol. 2026 Jan 16;16:1745319. doi: 10.3389/fimmu.2025.1745319 (PMC12855080; doi:10.3389/fimmu.2025.1745319)
Supplement: Supplementary file 1 [file DataSheet1.pdf]

Supplementary Material

Intranasal vaccines adjuvanted with Nexavant demonstrate robust protective efficacy by inducing both mucosal and systemic immunity in a murine model

Kwang Hyun Ko[1], Hyun Shik Bae[1], So Min Lee[1], Somin Park[2], Seung Hyun Han[2], Jun Heo[3], Jinil Kim[4], Yerim Cho[4], Dong-Ho Kim[1*], and Seung Bin Cha[1*]

1. Supplementary Table

Supplementary Table 1. List of mouse antibodies used in this study

Supplementary Table 2. Primer sequence information for qRT-PCR

Supplementary Table 3. Comparative summary of GLP toxicity studies of NVT administered via intranasal and intramuscular routes in NZW rabbits

2. Supplementary figures and figure legends

Supplementary Figure 1. Gating strategy used to analyze innate immune cell populations.

Supplementary Figure 2. Gating strategy used to analyze T cell responses.

Supplementary Figure 3. Increased intranasal volume may enhance systemic immunity due to potential lung delivery.

Supplementary Table 1. List of mouse antibodies used in this study

Antibody	Fluorochrome	Clone	Purpose	Vendor	Cat no.
Viability dye	eFluor780	-	Flow cytometry	eBioscience	65-0865-14
mCD11b	PE/Cyanine7	M1/70	Flow cytometry (For innate cells)	BioLegend	101216
mCD11c	APC	N418		BioLegend	117310
mF4/80	PE	BM8		BioLegend	123110
mLy6G	PerCP/Cyanine5.5	1A8		BioLegend	127616
mMHC-II	PE/Cyanine7	AF6-120.1		BioLegend	116420
mCD40	FITC	3/23	Flow cytometry (For DC activation)	BioLegend	124607
mCD80	PE	16-10A1		BioLegend	104708
mCD86	PE/Cyanine5	GL-1		BioLegend	105016
mCD4	PE/Cyanine7	RM4-5	Flow cytometry (For T-cell analysis)	BioLegend	100528
mCD8a	FITC	53-6.7		BioLegend	100706
mCD44	APC	IM7		BioLegend	103011
mIFN-γ	PE	XMG1.2		BioLegend	505808
mIgG-HRP	-	-	ELISA	SouthernBiotech	1030-05
mIgA-HRP	-	-		SouthernBiotech	1040-05

Supplementary Table 2. Primer sequence information for qRT-PCR

Primer name	Sequence (5′→3′)
TLR3	Forward: GAAGATGATGCAGTCTTTCCA<br>Reverse: CCTGTATCATATTCTACTCCTTGC
RIG-I	Forward: CAGACAGATCCGAGACACTA<br>Reverse: TGCAAGACCTTTGGCCAGTT
MDA-5	Forward: CGATCCGAATGATTGATGCA<br>Reverse: AGTTGGTCATTGCAACTGCT
IFN-β	Forward: AATGGAAAGATCAACCTCAC<br>Reverse: AAGGCAGTGTAACTCTTCTG
HPRT	Forward: CAATGCAAACTTTGCTTTCC<br>Reverse: CAAATCCAACAAAGTCTGGC

Supplementary Table 3. Comparative summary of GLP toxicity studies of NVT administered via intranasal and intramuscular routes in NZW rabbits

Test	Route	Regimen	Results
Single Dose Toxicity Study	Intramuscular	A single intramuscular injection of NVT at a dose of 8 mg/animal was administered to male and female NZW rabbits aged 17 weeks.	No mortality was observed; however, decreased appetite and slight body weight loss were noted.<br>The Maximum Tolerated Dose (MTD) was established at 8 mg/head.
	Intranasal	A single intranasal instillation of NVT at doses of 2, 4, or 8 mg/animal was administered to male and female NZW rabbits aged 18 weeks.	No mortality was observed.<br>The MTD was established at 8 mg/head.
Repeated Dose Toxicity Study (2-Week Dosing Period)	Intramuscular	NVT was administered via intramuscular injection at doses of 2, 4, or 8 mg/animal once weekly for 2 weeks to male and female NZW rabbits aged 17 weeks.	No mortality was observed in any dose group.<br>Injection site muscle cell necrosis and granulomatous inflammation were noted in both male and female animals at 4 mg/animal, and in males at 8 mg/head.<br>It is recommended that the group administered 8 mg/animalbe designated as the high-dose group in the 4-week repeated dose toxicity study.
	Intranasal	NVT was administered via intranasal instillation at doses of 2, 4, or 8 mg/animal twice weekly for 2 weeks to male and female NZW rabbits aged 17 weeks.	No mortality was observed in any dose group.<br>In the nasal administration site of both male and female NZW rabbits receiving 8 mg/animal, infiltration of multinucleated leukocytes was observed in the epithelium and lamina propriaof the nasal turbinate.<br>It is recommended that the group administered 8 mg/animal be designated as the high-dose group in the 4-week repeated dose toxicity study.
Repeated Dose Toxicity Study (4-Week Dosing Period and 2-Week Recovery Period)	Intramuscular	NVT was administered via intramuscular injection at doses of 0.3, 1, or 3 mg/animal once weekly for 4 weeks to male and female NZW rabbits aged 16 to 17 weeks, followed by a 2-week recovery period.	One mortality was observed in male NZW rabbits in each of the 1.0 and 3.0 mg/animal dose groups.<br>The No Observed Adverse Effect Level (NOAEL) could not be determined for male NZW rabbits. For female NZW rabbits, the NOAEL was determined to be 0.3 mg/head.
	Intranasal	NVT was administered via intranasal instillation at doses of 2, 4, or 8 mg/animal twice weekly for 4 weeks to male and female NZW rabbits aged 18 weeks, followed by a 2-week recovery period.	No mortality was observed in any dose group.<br>No systemic toxicity or target organ toxicity was observed.<br>The NOAEL was concluded to be 8 mg/head in both male and female animals.

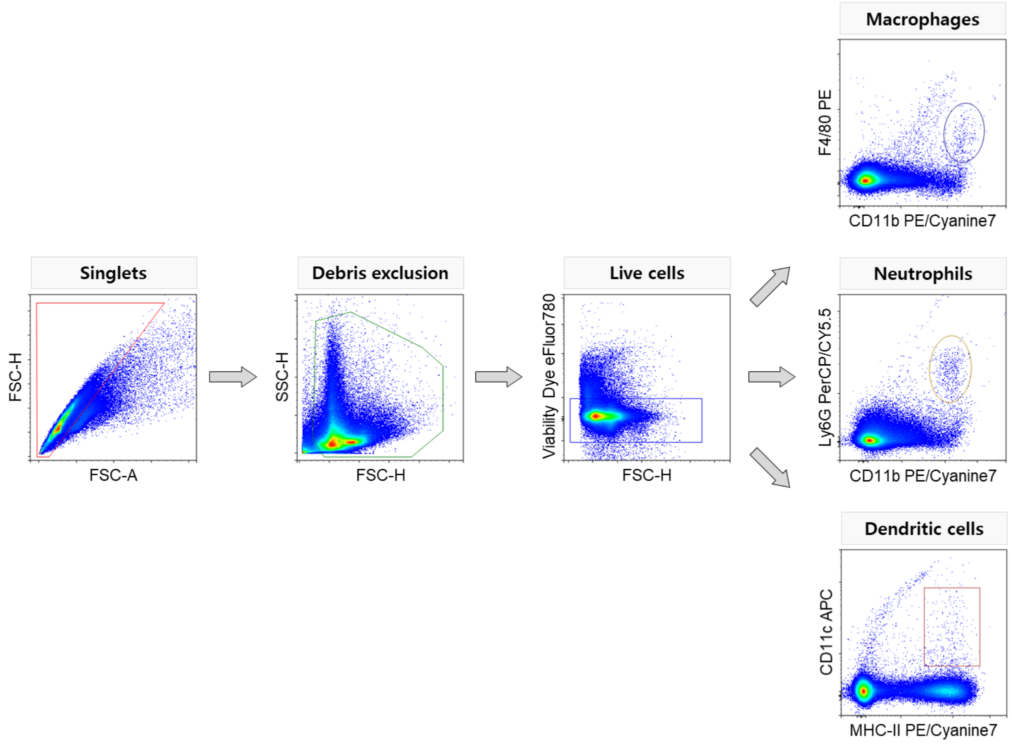

Supplementary Figure 1. Gating strategy used to analyze innate immune cell populations. Single cells were first gated based on FSC-A versus FSC-H to exclude doublets. Debris was removed by excluding events with low SSC-H and FSC-H values. Live cells were identified by excluding dead cells stained with a fixable viability dye. Macrophages were defined as $CD11b^{+}F4/80^{+}$, neutrophils as $CD11b^{+}Ly6G^{+}$, and dendritic cells as $CD11c^{+}MHC\text{-}II^{+}$.

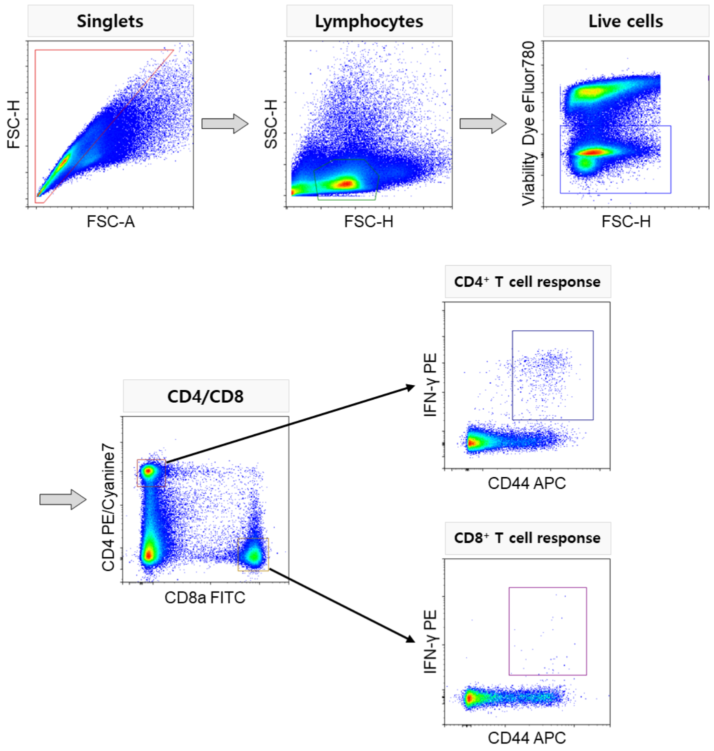

Supplementary Figure 2. Gating strategy used to analyze T cell responses. T cell responses were analyzed by first selecting singlets (FSC-A vs. FSC-H), then gating lymphocytes based on FSC and SSC. Live cells were identified using a viability dye. $CD4^{+}CD8^{-}$ cells were classified as $CD4^{+}$ T cells, and $CD4^{-}CD8^{+}$ cells were classified as $CD8^{+}$ T cells. Within each subset, $CD44^{+}$IFN-γ^{+} cells were analyzed.

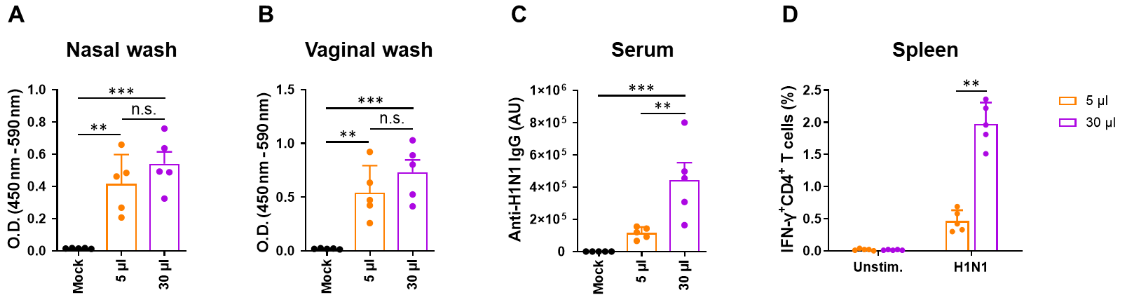

Supplementary Figure 3. Increased intranasal volume may enhance systemic immunity due to potential lung delivery. C57BL/6 mice (n = 5 per group) were intranasally immunized with 10 µl or 30 µl of H1N1+NVT twice at a 3-week interval. Three weeks after the final immunization, spleen, serum, nasal wash, and vaginal wash samples were collected. **(A, B)** H1N1-specific IgA levels in nasal washes **(A)** and vaginal washes **(B)**. **(C)** H1N1-specific serum IgG levels. **(D)** H1N1-specific IFN-γ^{+}CD4^{+} T cell responses in the spleen.
